# Systemic analyses of expression patterns and clinical features for GIMAPs family members in lung adenocarcinoma

**DOI:** 10.18632/aging.103836

**Published:** 2020-10-28

**Authors:** Sisi Deng, Zhi Zhang, Xiaoli Lu, Qi Zhou, Shilin Xia, Mi Li

**Affiliations:** 1 Cancer Center, Union Hospital, Tongji Medical College, Huazhong University of Science and Technology, Wuhan 430022, P.R. China; 2 Institute (College) of Integrative Medicine, Dalian Medical University, Dalian 116044, P.R. China; 3 Clinical Laboratory of Integrative Medicine, The First Affiliated Hospital of Dalian Medical University, Dalian 116011, P.R. China; 4 Department of Palliative Medicine, Graduate School of Medicine, Juntendo University, Tokyo 1138421, Japan; 5 Department of Orthopedics, Tongji Hospital, Tongji Medical College, Huazhong University of Science and Technology, Wuhan 430030, P.R. China

**Keywords:** lung adenocarcinoma, GIMAPs, public databases, prognostic value

## Abstract

GTPase of immunity-associated proteins (GIMAPs) are frequently prescribed as important components of immune regulation complexes, which were known to play key roles in lung adenocarcinoma. However, little is known about the function of distinct GIMAPs in lung adenocarcinoma. To address this issue, this study investigated the biological function and pathway of GIMAPs in lung adenocarcinoma using multiple public databases. Absent expression of GIMAPs was found in lung adenocarcinoma at mRNA and protein levels. While a purity-corrected value uncovered that all GIMAPs were positively associated with the immune infiltration of lung adenocarcinoma. Furthermore, the expressions of GIMAPs were considered to be negatively associated with clinical cancer stages, patient’s gender and pathological tumor grades in patients with lung adenocarcinoma. Besides, higher mRNA expression of GIMAPs was significantly associated with longer overall survival of patients with lung adenocarcinoma. Taken together, these results may enable GIMAPs family members as diagnostic and survival biomarker candidates or even potential therapeutic targets for patients with lung adenocarcinoma.

## INTRODUCTION

Lung adenocarcinoma (LUAD) is the most frequent histologic subtype of lung cancer, which is the leading cause of cancer-related mortality worldwide [1]. Early diagnosis of this malignancy is still an optimal opportunity, which is regarded to be a fundamental basis of further therapies, such as targeted and immune-based therapies [2]. Unlike lung squamous cell carcinoma, LUAD mainly occurs and develops within terminal bronchioles and alveoli, which are hardly accessible by bronchoscopy. Therefore, the early-stage of LUAD has been more elusive and difficult to identify [3–5]. A better understanding of the underlying pathogenesis of LUAD would put forward a new insight into discovering the early-stage diagnostic biomarkers to provide an advanced therapeutic strategy for patients with LUAD. Due to the anatomical structure of LUAD, the immune contexture is very complex. Emerging evidence shows that tumor microenvironment impacts LUAD progression and clinical outcome [6]. The tumor microenvironment consists of diverse immune cells, including macrophage and lymphocyte. It has previously been observed that GTPase of immunity-associated proteins (GIMAPs) has been implicated in the regulation of cell survival for lymphomyeloid cells and can be detected highest in lymphoid organs [7]. To date, the GIMAPs cluster are reported on Chromosome 7q36.1, within 300 kb, including eight functional genes and one pseudogene (GIMAP3, also named GIMAP3P). The six genes GIMAP1, GIMAP2, GIMAP4, GIMAP5, GIMAP6, and GIMAP7 encode 33-46 kDa proteins with one GTP-binding domain, whereas GIMAP8 encodes a 75 kDa protein with three GTP-binding domains. GIMAP1-GIMAP5 is a readthrough transcript variant between the neighboring GIMAP1 and GIMAP5, and it encodes a fusion protein that shares sequence identity with each gene product [8]. GIMAPs family members are reported to implicated in the progression of cancer via a regulation of immunologic microenvironment.

**Figure 1:**
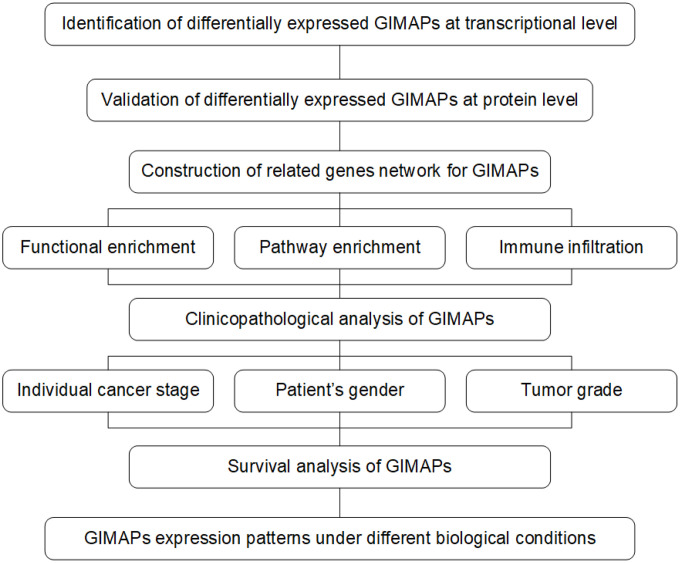
**The pipeline of this study based on public databases and platforms.**

Despite growing evidence in understanding the potential importance of GIMAPs in cancer, detailed mechanisms regarding the biological signature of GIMAPs in LUAD await elucidation. Exploring the molecular mechanism of GIMAPs has thus become imperative for advancing diagnosis and therapy of LUAD. In this study, we addressed this problem by a pipeline of systematic analysis (Figure 1), in order to identify the transcriptional and protein expression patterns of GIMAPs family members upon TCGA, Oncomine, and Human Protein Atlas databases. Then we continued to predict biological functions and pathways of GIMAPs as well as their 20 related genes. Subsequently, we analyzed clinical features and prognostic values of GIMAPs family members. Furthermore, we performed an analysis of molecular-disease association between GIMAPs and multiple disorders. The current study links the biological functionality and prognostic value of GIMAPs in LUAD, which will be beneficial to the diagnosis and treatment of lung adenocarcinoma.

## RESULTS

### Absent expression of GIMAPs family members in patients with LUAD

**Figure 2:**
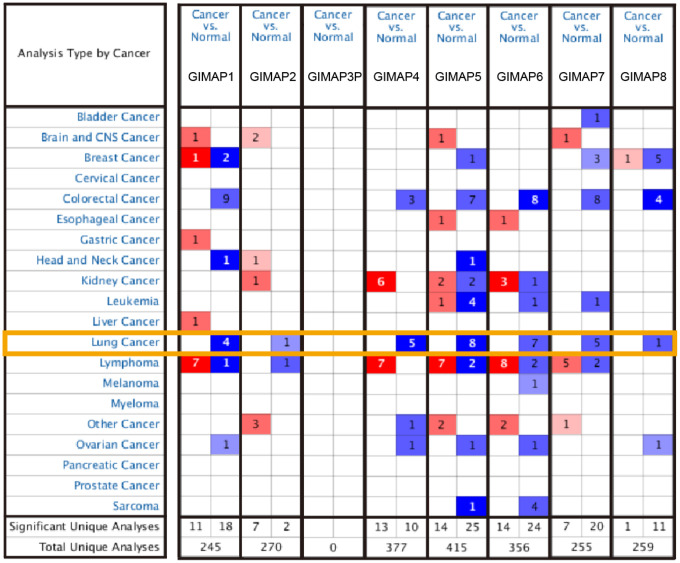
**Transcriptional expression of GIMAPs in 20 different types of cancer diseases (Oncomine database).** The difference in transcriptional expression was compared by t-test. The threshold statistical parameters were that, *p* value< 0.0001, fold change = 2, gene rank = 10%, and data type was mRNA.

**Figure 3:**
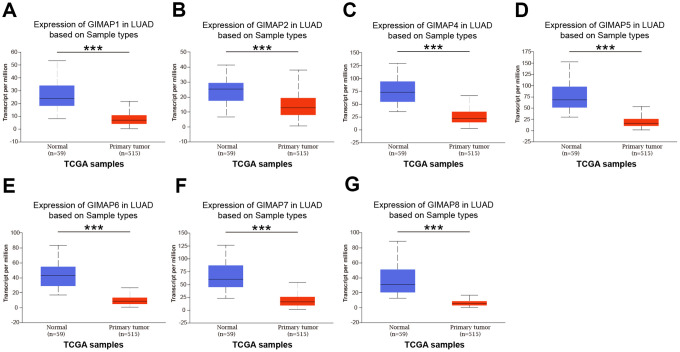
**mRNA expression of distinct GIMAPs in LUAD tissues and adjacent normal lung tissues (UALCAN).** mRNA expressions of GIMAPs family members were found to be under-expressed in primary LUAD tissues compared to normal samples (**A**–**G**). *** *p*<0.001.

In order to reveal the different potential therapeutic value of GIMAPs in LUAD patients, the mRNA expression of GIMAPs in 20 types of cancers were initially measured and compared to normal tissues by Oncomine database (Figure 2, Table 1). The under- expression of GIMAP1/2/4/5/6/7/8 were found in lung cancer tissues consistently, while neither GIMAP3 nor GIMAP1-GIMAP5 presented available data. In Okayama Lung dataset, GIMAP1 under-expression was found in LUAD tissues compared with normal tissues with a fold change of -3.359 (*p*=1.37E-13) [9], while Hou observed a 2.848-fold decrease in GIMAP1 mRNA expression in LUAD samples (*p*=4.73E-18) [10]. Similarly, significant down-regulation of GIMAP4 was also found in Selamat Lung [11], Okayama Lung [9], Hou Lung [10] database. GIMAP5 was also found in Su Lung [12], Selamat Lung [11], Okayama Lung [9], Hou Lung [10] database. GIMAP6 was also found in Landi Lung [13], Su Lung [12], Okayama Lung [9], Hou Lung [10] database. GIMAP7 was also found in Selamat Lung [11], Okayama Lung [9], Hou Lung [10] database. GIMAP8 was also found in Selamat Lung [11] database. However, GIMAP2 was found in the large cell lung carcinoma not in the LUAD with lower mRNA expression. Next, the mRNA expression patterns of GIMAPs were further validated by UALCAN using the resource based on RNA-seq from TCGA database. The mRNA expressions of other GIMAPs members were all found to be significantly down-regulated in LUAD tissues compared to normal samples (Figure 3).

**Table 1:** Significant changes of GIMAPs expression in transcription level between LUAD and normal lung tissues (ONCOMINE).

**GIMAPs**	**Types of LUAD VS. Lung**	**Fold Change**	**P-value**	**t-test**	**Ref**
GIMAP1					
	Lung Adenocarcinoma	-3.359	1.37E-13	-12.857	Okayama Lung
	Lung Adenocarcinoma	-2.848	4.73E-18	-10.665	Hou Lung
GIMAP4					
	Lung Adenocarcinoma	-3.652	2.77E-29	-16.284	Selamat Lung
	Lung Adenocarcinoma	-2.921	2.13E-08	-6.564	Su Lung
	Lung Adenocarcinoma	-2.02	4.1E-09	-6.72	Hou Lung
GIMAP5					
	Lung Adenocarcinoma	-2.926	5.8E-13	-9.931	Su Lung
	Lung Adenocarcinoma	-3.536	1.29E-31	-16.428	Selamat Lung
	Lung Adenocarcinoma	-2.41	4.38E-12	-10.856	Okayama Lung
	Lung Adenocarcinoma	-2.382	4.81E-13	-8.947	Hou Lung
GIMAP6					
	Lung Adenocarcinoma	-3.755	5.99E-25	-13.546	Landi Lung
	Lung Adenocarcinoma	-4.357	1.08E-10	-7.802	Su Lung
	Lung Adenocarcinoma	-2.894	1.84E-13	-12.45	Okayama Lung
	Lung Adenocarcinoma	-2.906	9.91E-14	-9.448	Hou Lung
GIMAP7					
	Lung Adenocarcinoma	-2.898	1.01E-17	-10.238	Selamat Lung
	Lung Adenocarcinoma	-2.495	1.21E-11	-10.53	Okayama Lung
	Lung Adenocarcinoma	-2.674	4.32E-12	-8.208	Hou Lung
GIMAP8					
	Lung Adenocarcinoma	-3.113	1E-27	-15.438	Selamat Lung

LUAD: Lung Adenocarcinoma; GIMAP: GTPase of immunity-associated protein.

**Figure 4:**
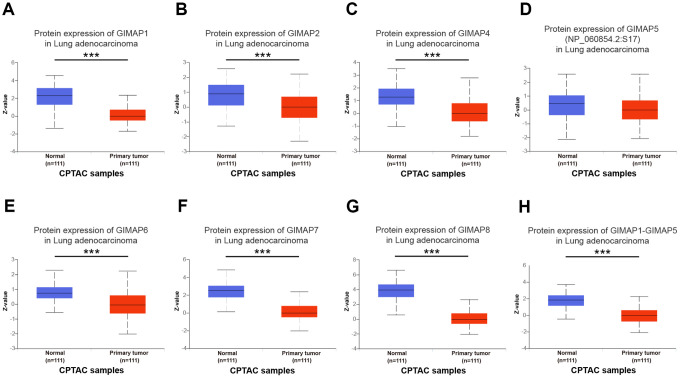
**Protein expression of distinct GIMAPs in LUAD tissues and adjacent normal lung tissues (UALCAN).** Protein expressions of GIMAPs family members were found to be under-expressed in primary LUAD tissues compared to normal samples (**A**–**H**). *** *p*<0.001.

**Figure 5:**
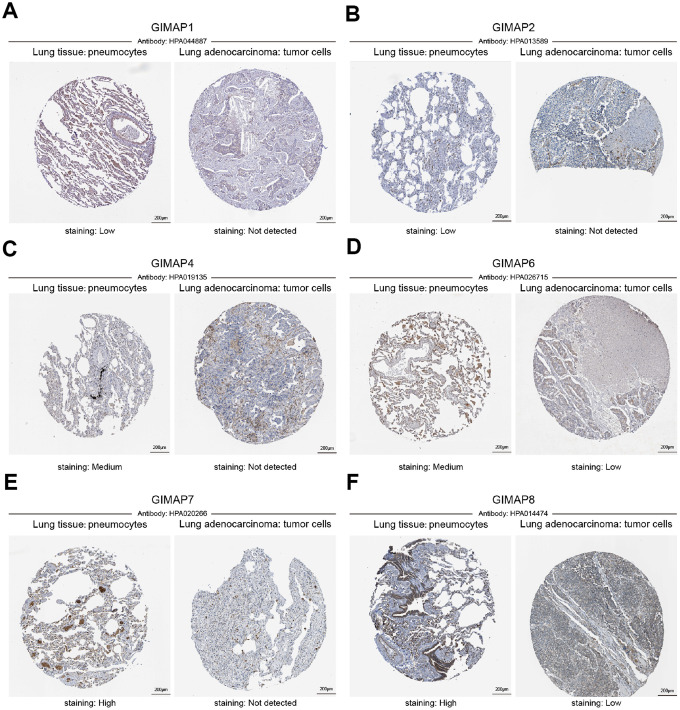
**Representative immunohistochemistry images of distinct GIMAPs in LUAD tissues and normal lung tissues (Human Protein Atlas).** GIMAP1/2 proteins were low expressed in normal lung tissues, whereas not expressions of them were observed in LUAD tissue (**A**, **B**). In addition, medium protein expressions of GIMAP4/6 were expressed in normal lung tissues, while not and low protein expressions of them were observed in LUAD tissue (**C**, **D**). Moreover, GIMAP7/8 would have high protein expressions in normal lung tissues compared with the LUAD tissue have not and low protein expressions (**E**, **F**).

Furthermore, the protein expression patterns of GIMAPs were examined to describe gene expression from protein level which is closer to the most primitive form of the disease (Figure 4). Firstly, the protein expression patterns of GIMAPs were measured by UALCAN using the resources based on Clinical Proteomic Tumor Analysis Consortium (CPTAC) database. Unfortunately, there was only phosphoprotein not total protein data of GIMAP5, and the protein expression has no significant difference, while other GIMAPs have a high significant difference. Next, the Human Protein Atlas was used to explore the immunohistochemistry images of GIMAPs. GIMAP1/2 proteins were low expressed in normal lung tissues, whereas not expressions of them were observed in LUAD tissue (Figure 5A, 5B). Also, medium protein expressions of GIMAP4/6 were expressed in normal lung tissues, while not or low protein expressions of them was observed in LUAD tissue (Figure 5C, 5D). Moreover, GIMAP7/8 would have high protein expressions in normal lung tissues compared with that in LUAD sample (Figure 5E, 5F).

Generally, all the results above showed that transcriptional and protein expressions of GIMAPs were under-expressed in patients with LUAD.

### Predicted functions and pathways of GIMAPs in LUAD patients

**Figure 6:**
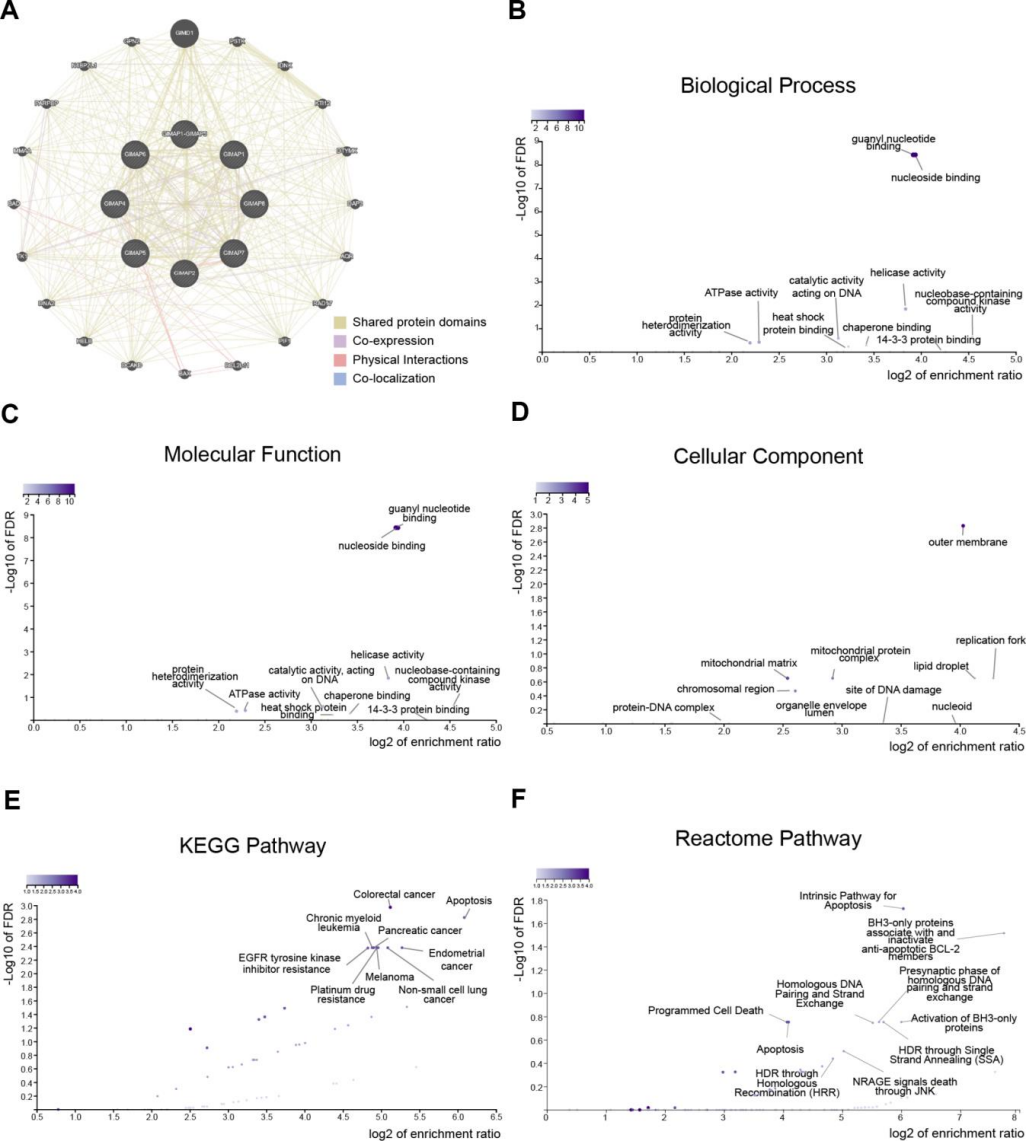
**Predicted functions and pathways of GIMAPs and their 20 related genes in LUAD patients (GeneMANIA and WebGestalt).** Network of GIMAPs and their 20 related genes was constructed (**A**). GO functional enrichment analysis predicted three main functions of GIMAPs and their 20 related genes, including biological process, cellular components and molecular functions (**B**–**D**). KEGG and Reactome pathway analysis on GIMAPs and their 20 related genes was shown at figure (**E**, **F**).

To explore the biological function of GIMAPs, we constructed a network of GIMAPs and their 20 related genes by GeneMANIA (Figure 6A). Most of the related genes were in the category named shared protein domains, which means two gene products are linked if they have the same protein domain.

Moreover, GO functions and pathways of GIMAPs and their 20 related genes were analyzed by WEB-based Gene SeT AnaLysis Toolkit (WebGestalt). The biological processes such as GO:0051052 (regulation of DNA metabolic process), GO:0038034 (signal transduction in absence of ligand), GO:0090559 (regulation of membrane permeability), and GO:0006260 (DNA replication) were remarkably regulated by the GIMAPs in LUAD (Figure 6B and Table 2). Cellular components, including GO:0019867 (outer membrane), GO:0005759 (mitochondrial matrix), GO:0005657 (replication fork), GO:0005811 (lipid droplet) and GO:0098798 (mitochondrial protein complex) were significantly associated with the GIMAPs alterations (Figure 6C). Besides, GIMAPs also prominently involved in the molecular functions, such as GO:0001882 (nucleoside binding), GO:0019001 (guanyl nucleotide binding), GO:0004386 (helicase activity), GO:0019205 (nucleobase-containing compound kinase activity) and GO:0140097 (catalytic activity, acting on DNA) (Figure 6D).

**Table 2:** The top 5 enriched GO terms of the GIMAPs and their 20 related genes in LUAD patients (WebGestalt).

**Category**	**GO ID**	**GO term**	**Count**	**FDR**	**Log P**	**Ratio**
Biological Process	GO:0051052	regulation of DNA metabolic process	405	0.0048132	-5.25	12.188
Biological Process	GO:0038034	signal transduction in absence of ligand	69	0.031887	-4.12	35.771
Biological Process	GO:0090559	regulation of membrane permeability	79	0.031887	-3.95	31.243
Biological Process	GO:0006260	DNA replication	268	0.04701	-3.58	12.279
Biological Process	GO:0071887	leukocyte apoptotic process	107	0.04701	-3.56	23.067
Cellular Component	GO:0019867	outer membrane	206	0.024847	-3.84	13.979
Cellular Component	GO:0005759	mitochondrial matrix	462	0.18341	-2.52	6.2331
Cellular Component	GO:0005657	replication fork	69	0.18341	-2.4	20.867
Cellular Component	GO:0005811	lipid droplet	77	0.18341	-2.31	18.699
Cellular Component	GO:0098798	mitochondrial protein complex	266	0.18341	-2.27	8.1195
Molecular Function	GO:0001882	nucleoside binding	384	0.914081	-12.15	16.745
Molecular Function	GO:0019001	guanyl nucleotide binding	390	1.19624	-12.07	16.487
Molecular Function	GO:0004386	helicase activity	150	3.348849	-3.81	14.289
Molecular Function	GO:0019205	nucleobase-containing compound kinase activity	46	4.30035	-2.48	23.297
Molecular Function	GO:0140097	catalytic activity, acting on DNA	184	11.86587	-2.33	8.7363

In KEGG analysis, these pathways including hsa01521 (EGFR tyrosine kinase inhibitor resistance), hsa05210 (colorectal cancer), hsa04210 (apoptosis), hsa05014 (amyotrophic lateral sclerosis) and hsa05213 (endometrial cancer) were associated with the functions of GIMAPs in LUAD (Figure 6E and Table 3). While in the Reactome analysis, the most significantly enriched terms were involved in R-HSA-109606 (intrinsic pathway for apoptosis), R-HSA-111453 (BH3-only proteins associate with and inactivate anti-apoptotic BCL-2 members), R-HSA-114452 (activation of BH3-only proteins), R-HSA-109581 (apoptosis), and R-HSA-5357801 (programmed cell death) (Figure 6F and Table 4).

**Table 3:** The top 5 enriched KEGG pathway terms of the GIMAPs and their 20 related genes in LUAD patients (WebGestalt).

**Pathway ID**	**Pathway name**	**Count**	**FDR**	**Log P**	**Ratio**
hsa01521	EGFR tyrosine kinase inhibitor resistance	79	0.019198	-4.04	31.515
hsa05210	Colorectal cancer	86	0.019198	-3.93	28.95
hsa04210	Apoptosis	136	0.049746	-3.34	18.306
hsa04215	Apoptosis	33	0.05448	-3.17	50.296
hsa05014	Amyotrophic lateral sclerosis	51	0.10407	-2.8	32.545

**Table 4:** The top 5 Reactome terms of the GIMAPs and their 20 related genes in LUAD patients (WebGestalt).

**Pathway ID**	**Pathway name**	**Count**	**FDR**	**Log P**	**Ratio**
R-HSA-109606	Intrinsic Pathway for Apoptosis	44	0.018834	-4.96	65.417
R-HSA-111453	BH3-only proteins associate with and inactivate anti-apoptotic BCL-2 members	9	0.030598	-4.45	213.21
R-HSA-114452	Activation of BH3-only proteins	30	0.1769	-3.37	63.964
R-HSA-109581	Apoptosis	169	0.1769	-3.22	17.032
R-HSA-5357801	Programmed Cell Death	172	0.1769	-3.2	16.735

**Figure 7:**
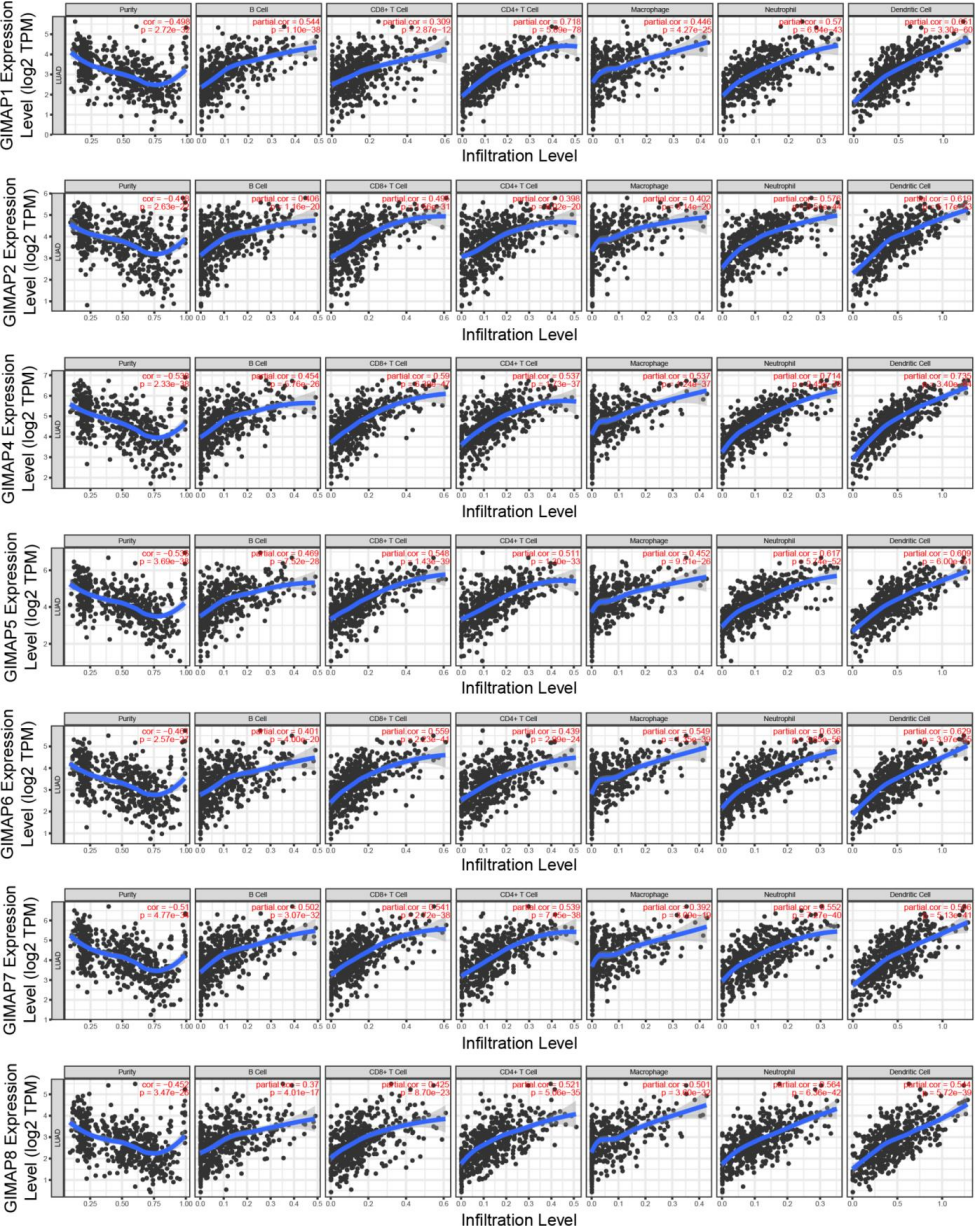
**Immune infiltration analysis of GIMAPs family members (TIMER).** The purity-corrected value of all GIMAPs were negative, that means these genes were highly expressed in the microenvironment.

After GO and pathway analyzing, immune infiltration of GIMAPs was estimated by Tumor IMmune Estimation Resource (TIMER). The scatterplots of GIMAPs showed a purity-corrected value and statistical significance. All GIMAPs had negative associations with tumor purity, which means these genes were highly involved in the progression of immune infiltration in LUAD (Figure 7 and Table 5).

**Table 5:** Correlation coefficient of immune infiltration level with GIMAPs expression level (TIMER).

	**Purity**	**B cell**	**CD8+ T cell**	**CD4+ T cell**	**Macrophage**	**Neutrophil**	**Dendritic cell**
GIMAP1	-0.4989 (*p* = 2.72e-32)	0.544 (*p* = 1.10e-38)	0.309 (*p* = 2.87e-12)	0.718 (*p* = 5.69e-78)	0.446 (*p* = 4.27e-25)	0.57 (*p* = 6.04e-43)	0.651 (*p* = 3.30e-60)
GIMAP2	-0.418 (*p* = 2.63e-22)	0.406 (*p* = 1.16e-20)	0.495 (*p* = 1.56e-31)	0.398 (*p* = 8.02e-20)	0.402 (*p* = 3.14e-20)	0.576 (*p* = 5.51e-44)	0.619 (*p* = 5.17e-53)
GIMAP4	-0.538 (*p* = 2.33e-38)	0.454 (*p* = 5.76e-26)	0.59 (*p* = 6.29e-47)	0.537 (*p* = 1.73e-37)	0.537 (*p* = 1.24e-37)	0.714 (*p* = 2.45e-76)	0.735 (*p* = 3.40e-84)
GIMAP5	-0.538 (*p* = 3.69e-38)	0.469 (*p* = 7.52e-28)	0.548 (*p* = 1.43e-39)	0.511 (*p* = 1.30e-33)	0.452 (*p* = 9.51e-26)	0.617 (*p* = 5.74e-52)	0.609 (*p* = 6.00e-51)
GIMAP6	-0.461 (*p* = 2.57e-27)	0.401 (*p* = 4.00e-20)	0.559 (*p* = 2.23e-41)	0.439 (*p* = 2.99e-24)	0.549 (*p* = 1.35e-39)	0.636 (*p* = 3.65e-56)	0.629 (*p* = 3.97e-55)
GIMAP7	-0.51 (*p* = 4.77e-34)	0.502 (*p* = 3.07e-32)	0.541 (*p* = 2.72e-38)	0.539 (*p* = 7.45e-38)	0.392 (*p* = 3.09e-19)	0.552 (*p* = 7.27e-40)	0.566 (*p* = 5.13e-14)
GIMAP8	-0.452 (*p* = 3.47e-26)	0.37 (*p* = 4.01e-17)	0.425 (*p* = 8.70e-23)	0.521 (*p* = 5.06e-35)	0.501 (*p* = 3.60e-32)	0.564 (*p* = 6.36e-42)	0.544 (*p* = 5.72e-39)

Taken together, the results showed that GIMAPs played a potential promotive role in the regulation of the DNA metabolic process and involved in apoptosis, as well as immune infiltration.

### Association of transcriptional and protein expression of GIMAPs family members with clinicopathological features of LUAD patients

**Figure 8:**
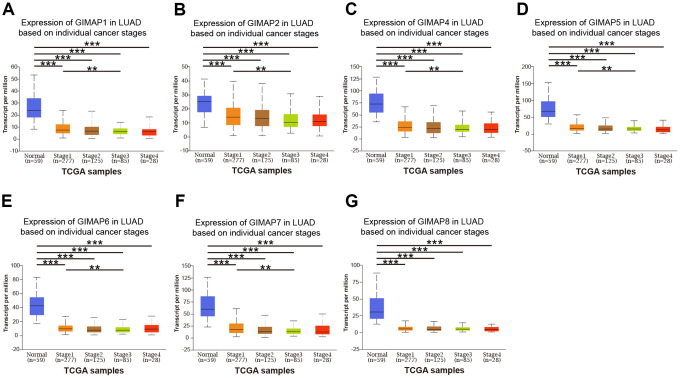
**Relationship between mRNA expression of distinct GIMAPs and individual cancer stages of LUAD patients (UALCAN).** mRNA expressions of GIMAPs family members were remarkably correlated with patients’ individual cancer stages, patients who were in more advanced cancer stages tended to express lower mRNA expression of GIMAPs (**A**–**G**). **p<0.01, ***p<0.001.

**Figure 9:**
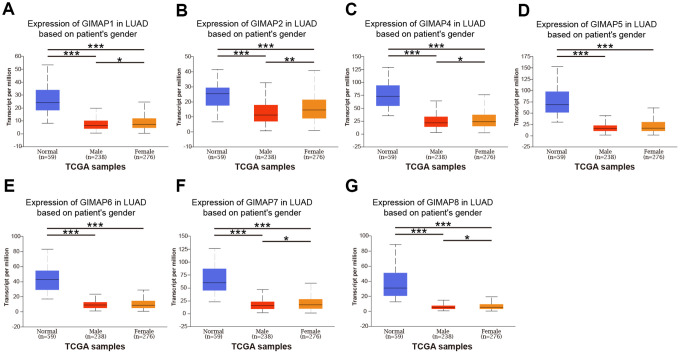
**Relationship between mRNA expression of distinct GIMAPs and patient’s gender of LUAD (UALCAN).** mRNA expressions of GIMAPs family members were remarkably correlated with patient’s gender, male patients tended to express lower mRNA expression (**A**–**G**). *p<0.05, **p<0.01, ***p<0.001.

**Figure 10:**
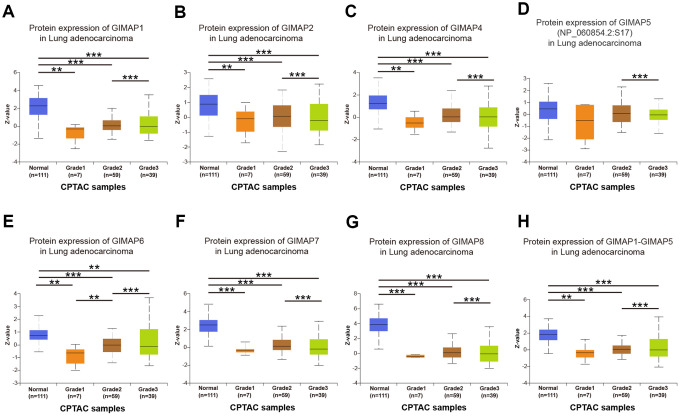
**Association of protein expression of distinct GIMAPs with tumor grades of LUAD patients (UALCAN).** protein expression of GIMAPs were significantly related to tumor grade, as tumor grade increased, the protein expression of GIMAPs tended to be higher (**A**–**H**). **p<0.01, ***p<0.001.

Further, we analyzed a relationship between GIMAPs’ expression with the clinicopathological characteristics of LUAD patients by UALCAN, including individual cancer stage, patient’s gender, and tumor grade. The mRNA expression of GIMAPs based on RNA-seq was from TCGA database and protein expression from CPTAC. The mRNA expressions of GIMAPs were remarkably correlated with patients’ individual cancer stages, and patients who were in more advanced cancer stages tended to express lower mRNA expression of GIMAPs (Figure 8). Similarly, mRNA expressions of GIMAPs were significantly related to patient’s gender, and male patients tended to express lower mRNA expression (Figure 9). Besides that, protein expression of GIMAPs were significantly related to tumor grade, as tumor grade increased, the protein expression of GIMAPs tended to be higher (Figure 10). In short, these results suggested that 8 GIMAPs family members were significantly associated with clinicopathological features in LUAD patients.

### Prognostic value of mRNA expression of GIMAPs in LUAD patients

**Figure 11:**
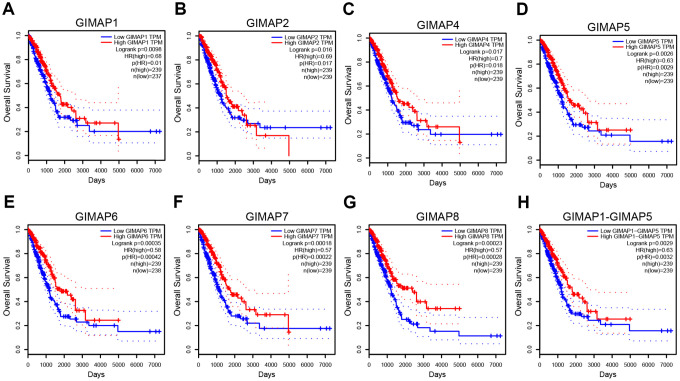
**Prognostic value of mRNA expression of distinct GIMAPs in LUAD patients (GEPIA).** mRNA expressions of all the GIMAPs family members were significantly associated with LUAD patients’ prognosis, higher mRNA expression of GIMAPs were significantly associated with longer overall survival of LUAD patients (**A**–**H**).

To analyze the prognostic value of GIMAPs in LUAD patients, we carried out a prognostic evaluation for the mRNA expression of GIMAPs in LUAD patients by GEPIA. As the results showed, mRNA expressions of all GIMAPs family members were significantly associated with LUAD patients’ prognosis, higher mRNA expression of GIMAP1 (HR=0.68, and p=0.0098), GIMAP2 (HR=0.69, and p=0.016), GIMAP4 (HR=0.7, and p=0.017), GIMAP5 (HR=0.63, and p=0.0026), GIMAP6 (HR=0.58, and p=0.00035), GIMAP7 (HR=0.57, and p=0.00018), GIMAP8 (HR=0.57, and p=0.00023), GIMAP1-GIMAP5 (HR=0.63, and p=0.0029) were significantly associated with longer overall survival of LUAD patients (Figure 11). These results indicated that higher mRNA expressions of GIMAPs could increase the survival time of LUAD patients and they may be exploited as potentially useful biomarkers to predict the LUAD patients’ survival.

### The molecular-disease association of GIMAPs with multiple diseases

**Figure 12:**
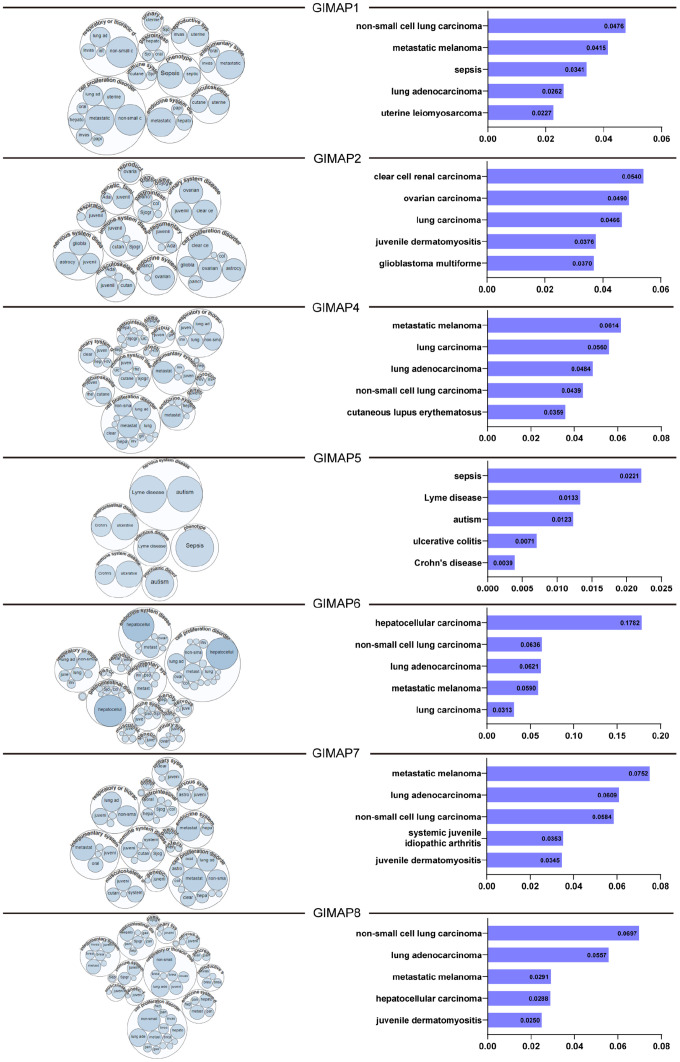
**Relationship between GIMAPs with LUAD among the different disease (the Open Targets Platform).** GIMAPs had a close relationship with LUAD and could be determined and visualized. The bubbles color and bubbles size represent the association score, and the top 5 diseases with a high score value are listed at right.

After analyzing the prognostic value of GIMAPs in LUAD patients, we further explore the relationship of GIMAPs with various diseases by the Open Targets Platform. GIMAP1/4/6/7/8 were predicted to have a higher association with LUAD filtered by RNA expression in this study, while GIMAP2 was predicted to have an association with lung carcinoma and GIMAP2 was predicted to have an association with sepsis (Figure 12). All in a word, GIMAPs had a close relationship with LUAD and they may be predicted as potential therapeutic targets for LUAD patients.

## DISCUSSION

In the tumor microenvironment, aberrant lymphocyte immune infiltration has been found to take part in the development and progression of LUAD [7]. Being important factors of regulation complexes for immune infiltration, dysregulation expression of GIMAPs family are implicated in the development of LUAD [14]. However, distinct roles of GIMAPs family members in LUAD remained to be elucidated. In this study, the transcriptional and protein expression of GIMAPs family members in LUAD were identified based on distinct public databases. Furthermore, the mRNA expression of GIMAPs was significantly associated with patients’ individual cancer stages and patient’s gender in LUAD patients, while the protein expression of GIMAPs was significantly associated with tumor grades in LUAD patients. Moreover, higher mRNA expressions of GIMAPs significantly associated with longer OS in LUAD patients. Finally, as in the target-disease relationship, GIMAPs had a close relationship with cell proliferation disorders, especially LUAD.

The functions enrichment of GIMAPs and their 20 related genes in LUAD patients were analyzed and our results showed that biological processes such as GO:0051052 (regulation of DNA metabolic process), cellular components such as GO:005657 (replication fork), and molecular functions such as GO:0001882 (nucleoside binding) were remarkably regulated by the GIMAPs and their 20 related genes in LUAD. Mechanistic studies *in vitro* reveal that impairment of GIMAP5-deficient TH cell differentiation is associated with increased DNA damage, which could be controlled by TGF-βin GIMAP5-deficient CD4+ T cells to induce TH17 polarization [15]. Previous *in vivo* study showed that GIMAP3/5 modified mitochondrial DNA in a heteroplasmic mouse model [16], which was consistent with our findings that GIMAPs family members influenced DNA metabolism in either mitochondria or nucleus.

In this study, KEGG pathways enrichment such as hsa05210 (colorectal cancer) and hsa05213 (endometrial cancer) indicated that GIMAPs mediated progression of various malignancy not only lung cancer. It was reported that the downregulation of the mRNA and protein expression levels of GIMAP5 and GIMAP6 in the tumor tissues and blood of patients with hepatocellular carcinoma. That suggested GIMAP5 and GIMAP6 could involve in the pathogenesis of hepatocellular carcinoma [17]. GIMAP6 had been reported as a one of the hub genes in associated with the pathogenesis of LUAD using the data from the Gene Expression Omnibus (GEO) database. GIMAP6 was expressed at lower levels in the tumor samples with a better prognosis [18], which is the same with our study. GIMAP7 was associated with the infiltration levels of immunocytes, and the inhibition of GIMAP7 would down-regulated the level of FOXO1 expression in pancreatic adenocarcinoma [19]. Mechanistically, GIMAP6 and GIMAP8 showed a reduction of gene expression in the tumors and GIMAP8 mRNA level was abnormally elevated in the adjacent nontumor tissues as compared, which was confirmed by quantitative PCR assays [14]. These studies accord with our implication that GIMAPs family members may play potent roles on tumorigenesis and progression of cancer.

According to Reactome analysis of R-HSA-109606/10958/5357801(apoptosis), our findings exhibited that GIMAPs were involved in regulation of apoptosis. Previous evidence has established that GIMAPs family members are important in regulating apoptosis in cancer cells [17]. GIMAP3 and GIMAP5 proteins seem to play essential roles in T cell homeostasis, via notably ability to improve the activity of Bcl-2 family members [20, 21]. Studies revealed GIMAP4 regulated apoptosis of T-cell by intrinsic stimuli downstream of caspase-3 activation and phosphatidylserine exposure. And the execution of programmed cell death directly correlates with the phosphorylation status of GIMAP4 [22]. Datta P et al. also reported that GIMAP1 ablation is accompanied by activation of the extrinsic apoptotic pathway [23]. Given that pathway and Reactome analyses of GIMAPs, it is likely that GIMAPs are involved in molecular pathogenesis of cancer via regulation of apoptosis. However, this hypothesis needs to be tested.

Among GIMAPs family members, GIMAP1/2/4/7/8 provided a significant difference between genders, which suggested a potential role of GIMAPs with sex hormone. Prior research based on RNA-seq noted a clue between GIMAPs and hormone [24]. But little is known regarding the depth molecular mechanism underlying a relationship between GIMAPs and hormone. In addition, clinical traits of LUAD patients were used to assess the clinical features of GIMAPs. We found that GIMAPs expression laid out a significance between groups with different stages as well as grades. From our findings of these clinical characteristics, GIMAPs family members were significantly associated with stage and grade of LUAD. The independent prognostic value of GIMAPs in LUAD pleas for a further understanding of mechanism by which GIMAPs family members drive tumorigenesis and development of lung adenocarcinoma.

Results from our study showed that lack of mRNA and protein expression was found in all the GIMAPs family members in LUAD, which suggested that lack of GIMAPs expressions would be one of predictors in LUAD. However, there is an academic report presented that aberrant activation of the GIMAP enhancer contributed to regulate the transcription factor TAL1/SCL, which was one of the most prevalent oncogenes in T-cell acute lymphoblastic leukemia [25]. Hence a further investigation would be conducted for exploring the molecular mechanism underlying a biological function of GIMAPs family members.

Obviously, there were some limitations in this study. First, all the data analyzed was based on bioinformatics from the online databases, further *in vivo* and *in vitro* studies are required to verify the biological functions of these findings. Second, we did not explore the underlying mechanisms of distinct GIMAPs in LUAD. Further fully designed experiments and analyses are worth to reveal the detailed mechanism between GIMAPs and LUAD. Finally, this study was only a retrospective study, and further prospective results are needed to support each other.

In conclusion, our results showed that under expression of GIMAPs members was found in the LUAD patients based on distinct public databases. GIMAPs were significantly associated with individual cancer stage, patient’s gender, and tumor grade. Besides, GIMAPs family members accompanied with immune infiltration of LUAD. Moreover, high mRNA expressions of GIMAPs were significantly related with longer OS in LUAD patients. All in a word, GIMAPs may become the potential diagnostic biomarkers and predicted as useful survival biomarkers for the LUAD patients.

## MATERIALS AND METHODS

### Identification of differentially expressed GIMAPs at transcriptional level

Oncomine (https://www.oncomine.org/resource/login.html) provides robust, peer-reviewed analysis methods and a powerful set of analysis functions to compute gene expression signatures [26]. In this study, Oncomine database is used to identify the transcriptional expressions of GIMAPs family members in LUAD tissues with their corresponding adjacent normal control samples. The data in our study was compared by t-test and the threshold statistical parameters were that, *p* value < 0.0001, fold change = 2, gene rank = 10%, and data type was mRNA.

UALCAN (http://ualcan.path.uab.edu) is a comprehensive and interactive web resource based on RNA-seq of 26 cancer types from TCGA database [27]. To determine the reliability of the differential expressed data, UALCAN database was chosen to further verify. In this study, the mRNA expressions of different GIMAPs between LUAD tissues with normal tissues were performed in the TCGA-LUAD dataset with a statically significant *p* < 0.001.

### Identification of differentially expressed GIMAPs at protein level

Besides the transcriptional analysis from TCGA, UALCAN database could provide protein expression analysis using data from CPTAC Confirmatory/Discovery dataset for LUAD [28]. The proteome-based subtypes could be observed in external cancer proteomic datasets. The protein expressions of GIMAPs family members between LUAD tissues with normal tissues were performed with a statically significant *p* < 0.001.

The Human Protein Atlas (https://www.proteinatlas.org) is a website that contains protein expression data for near 20 highly common kinds of cancers based on representative immunohistochemistry data [29]. In order to avoid the result bias caused by data from a single database source, the Human Protein Atlas was applied to identify the tumor-type specific proteins expression patterns of GIMAPs that are differentially expressed in LUAD patients. The immunohistochemistry images of GIMAPs expressed in LUAD and normal tissues could be directly visualized.

### Construction of related genes network and functional analysis

GeneMANIA (http://www.genemania.org) is a flexible web interface for generating hypotheses about gene function using available genomics and proteomics data [30]. In our study, the GIMAPs family members were submitted to the GeneMANIA to illustrate the functional association network among GIMAPs and their related genes. The advanced statistical options used were as follows: max resultant genes = 20, max resultant attributes = 10, using the automatically selected weighing method.

WebGestalt (http://www.webgestalt.org) is a functional enrichment analysis web tool with continuously updated and effectively reduce data redundancy [31]. GO functions and pathways of GIMAPs and their 20 related genes were enriched by WebGestalt. The Method of Interest is selected in Over-Representation Analysis (ORA). The GO functional enrichment was performed in the biological process no Redundant (BP), cellular component no Redundant (CC), molecular function no Redundant (MF). And the pathway analysis was performed in KEGG and Reactome pathway.

TIMER (https://cistrome.shinyapps.io/timer/) is a comprehensive web server using the microarray expression values for calculating a systematic analysis of immune infiltrates across diverse cancer types [32]. The immune infiltration estimation of GIMAPs was performed in LUAD by TIMER. The scatterplots of GIMAPs was generated to show the purity-corrected partial Spearman’s rho value and statistical significance. The positive purity value expected genes highly expressed in the tumor cells, and the opposite is expected for genes highly expressed in the microenvironment.

### Clinicopathological analysis of GIMAPs in LUAD

Furthermore, UALCAN was used to analyze the association between the mRNA or protein expressions of GIMAPs in LUAD tissues with their clinicopathologic parameters such as individual cancer stage, patient’s gender, and tumor grade. The results could be got directly by selecting the clinicopathological grouping options integrated into the UALCAN database. In particular, only the tumor group could be divided into different clinicopathological groups. The statically significant *p* is less than 0.001.

### Survival analysis of GIMAPs

The prognostic value of mRNA expression of distinct GIMAPs in LUAD was analyzed by using GEPIA (http://gepia.cancer-pku.cn/index.html) [33]. which could analyze the RNA sequencing expression data of 9,736 tumors and 8,587 normal samples from the TCGA and the GTEx projects. In this study, patients with LUAD were divided into high and low expression groups based on median values of mRNA expression. The statically significant difference was considered when a *p*-value < 0.05.

### GIMAPs expression patterns under different biological conditions

The Open Targets Platform (https://www.targetvalidation.org) is a comprehensive and powerful data integration for accessing and visualizing potential drug targets related to diseases [34]. To further explore the relationship between GIMAPs with LUAD among multiple diseases, the Open Targets Platform was used to show the available evidence for GIMAPs-disease associations. Data type was filtered by mRNA expression in this study. All methods and Strategies above could be seen in the flow chart below (Figure 1).
